# Novel Measles Virus Genotype, East Timor and Australia

**DOI:** 10.3201/eid0807.010409

**Published:** 2002-07

**Authors:** Doris Chibo, Michaela Riddell, Michael Catton, Christopher Birch

**Affiliations:** * World Health Organization Regional Measles Reference Laboratory for the Western Pacific Region, North Melbourne, Victoria, Australia

**Keywords:** Measles virus, novel genotype g3, East Timor, refugees, Australia

## Abstract

Measles outbreaks in 1999 in Queensland and Victoria, Australia, were caused by a novel strain of clade G virus (proposed name g3). Epidemiologic and molecular evidence supports independent circulation of this virus in Queensland, northern Australia, in addition to importation of the virus by East Timor refugees seeking safe haven in Australia.

Strategies to reduce *Measles virus* (MeV) circulation in industrialized countries, such as aggressive vaccination campaigns targeting children, have proven successful (1). Nevertheless, through introduction from other geographic locations, the virus continues to cause outbreaks in industrialized countries in unvaccinated persons.

In Victoria, measles is a reportable disease, and an enhanced surveillance strategy has been operational since 1997. A registered nurse visits the homes of patients with reported cases to collect specimens for laboratory confirmation, by polymerase chain reaction or detection of MeV-specific immunoglobulin (Ig) M, of the clinical diagnosis and subsequent identification of the measles genotype (6). For other Australian states, specimens from laboratory-confirmed cases are sent to the WHO regional measles reference laboratory for the western Pacific Region.

Variable regions in the MeV genome include the hemagglutinin (H) and nucleoprotein (N) genes. The most variable region is the carboxyl-terminal end (450 nucleotides [nt]) of the N gene. A uniform nomenclature approved by World Health Organization (WHO) has existed since 1998 and is used in classifying and naming measles viruses. Currently, 20 genotypes and 1 proposed new genotype exist, encompassing eight clades designated A–H. Each clade contains MeV genotypes that are related by >2.5% nt divergence in the 450-bp carboxyl-terminal end of the N gene and 2% in the H gene (2). Clades are distinguished by greater nucleotide differences, location of nucleotides, and specific nucleotides shared in genotypes of a particular clade.

Until recently, MeV strains belonging to clade G had not been detected for >15 years, and the lineage was considered to be either extinct or inactive (3). However, retrospective sequence analysis of a measles strain isolated from an immunocompromised infant from the Netherlands, who had been infected in Indonesia in 1997, and of measles strains associated with outbreaks in Indonesia and Malaysia in 1999 have demonstrated that this genotype has circulated in the intervening period (4–5). We describe the circulation of a novel genotype of MeV in Australia and investigate its likely origin.

## The Study

In June 1999, the novel genotype was first identified in Victoria in a 24-year-old Australian man who had symptoms of MeV infection shortly after visiting the northern state of Queensland. In the next 2 months, four more cases of infection with the same genotype were identified in Queensland. Epidemiologic links between these five cases could not be established.

In the second week of September 1999, refugees from the newly independent country of East Timor arrived in Darwin, Northern Territory, where they underwent preliminary medical examinations before being moved to a safe-haven refugee camp in regional Victoria. Several days after arriving at the safe haven, a 4-year-old girl had symptoms that matched the clinical case definition of measles infection (rash and cough with fever at onset of rash) (7). The diagnosis was confirmed serologically with an Enzygnost Anti-measles-virus/IgM kit (Dade Behring, Marburg, Germany). Active surveillance of all contacts of the initial patient and all residents reporting to the safe-haven medical center with symptoms suggestive of measles infection identified 11 other cases (4 laboratory confirmed) in children <13 years of age in the safe haven; a 26-year-old volunteer worker who worked at the accident and emergency department of the same hospital that admitted some of the infected refugees was also diagnosed with measles infection. Subsequent molecular analysis confirmed the volunteer’s infection as being caused by the same virus as the refugees. The last recognized case of this MeV genotype occurred in early November 1999, when a United Nations International Force East Timor (Interfet) soldier, who showed clinical symptoms in East Timor immediately before he was transferred to Darwin, was confirmed as being infected with MeV.

The commonality of the virus strains circulating in both Australia and East Timor was confirmed through analysis of MeV RNA (8). Analysis of the 456-bp carboxyl-terminal region of the N gene of the Victorian and Queensland samples yielded identical sequences in most cases, suggesting that these patients were infected with the same strain of MeV as the East Timorese. Nevertheless, no epidemiologic links were apparent between the Queensland cases and the subsequent cases in East Timorese refugees.

The N gene sequences of measles viruses identified from June to November 1999 were most closely related to the Amsterdam prototype G2 strain identified by de Swart et al. (4). Although phylogenetic analysis indicated that these sequences belonged within the clade G viruses, they differed from the G2 prototype strain by 12 (2.6%) nt and by deduction 6 (4%) amino acids (Figure). N gene sequences were obtained from 17 clinical samples. As a group, these 17 samples shared an amino acid (439K) in the N protein not previously seen in any other reference prototypes analyzed. Thirteen of these samples were identical in sequence (samples 1–13, **Table**). Three samples (samples 14–16, **Table**) differed by a single nucleotide, which resulted in another novel amino acid change, P456L. One sample (sample 17, **Table**) diverged by 2 nt, one silent and the other resulting in the same P456L change seen in samples 14–16. Differences seen in these 17 samples did not appear to be related to the geographic location of the cases.

**Figure:**
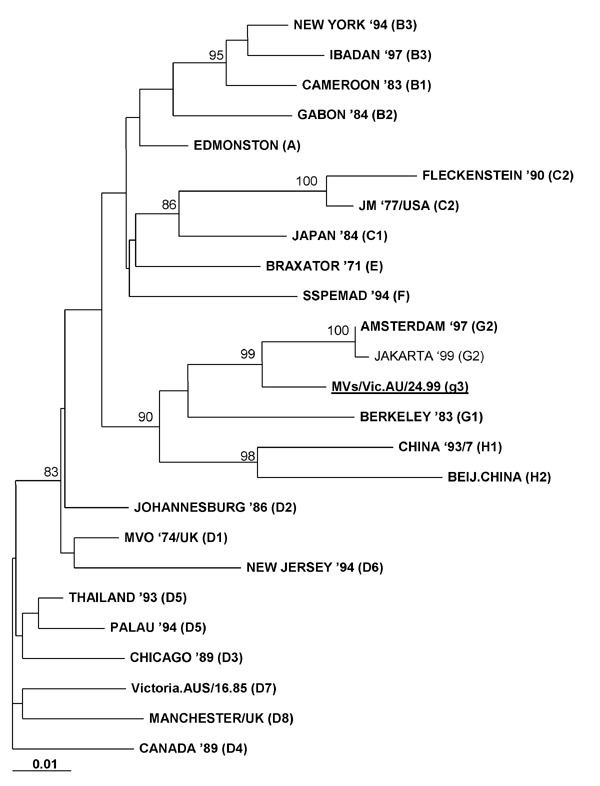
Phylogenetic analysis with the Phylip software program of DNAdist (maximum likelihood/neighbor-joining, 1000 bootstrap cycles) of the carboxyl-terminal 456-bp nucleoprotein (N) gene sequence of measles virus/Vic.AU/24.99 circulating in Australia and East Timor. World Health Organization–designated prototype strains are shown in bold, and the proposed new g3 genotype is shown in bold and underlined. Jakarta 1999 (G2) has also been included to show the difference between the clade G viruses. Statistically significant bootstrap values (>80%) are indicated. Scale (0.01) indicates nucleotide substitutions per site.

**Table:** Unique predicted amino acid differences compared with all reference measles virus sequences in 456-bp carboxyl-terminal end of N^a^ gene^b^

Sample no.	Predicted amino acid differences	Accession no.
1–13	439K^c^	AF353622
14–16	439K^c^; 456L	AY055850
17	439K^c^; 456L	AY055851

^a^N, nucleoprotein. ^b^Identified from 17 clinical samples in the outbreak ^c^Unique to proposed genotype g3.

Full or partial H gene sequences were derived from the measles viruses of four samples obtained from patients 8, 13, 15, and 16. Phylogenetic analysis of the H gene of the first 842 nt of these samples confirmed their assignment to the clade G viruses (results not shown). The four sequences analyzed showed up to 1.1% intranucleotide divergence. The MeV from patient 8 varied by 2.1% nt sequence compared with the prototype G2 strain. Comparison of nucleotide and amino acid sequence of the H gene of the one sample (from patient 13) with full H gene sequence showed differences from the prototype G2 strain of 35 (1.9%) nt and 13 (2.1%) amino acids. This sequence also contained amino acids in the H protein not previously found in other reference prototypes, namely 212Q, 225H, 238D, and 495N. Phylogenetic analysis of the full H gene sequence showed similar patterns of relatedness to those obtained for the N gene (results not shown).

Together, phylogenetic analysis of the N and H genes and the appearance of novel amino acids in the H protein provided strong evidence that these measles virus were sufficiently unrelated to the prototype G2 strain to enable their classification as a new genotype within clade G. We propose that measles virus/Vic.AU/24.99 be the reference sequence for a new genotype, g3, pending isolation of a reference strain (2). GenBank accession numbers for the 456-bp carboxyl-terminal end of three of the N gene sequences are shown in the Table. The GenBank accession number for the full-length H gene is AF35362.

The novel g3 MeV was not the only strain circulating in Australia during this time. Coinciding with the genotype g3 outbreak, four cases of genotype D8 were identified in Victoria. Three months earlier, small clusters of measles cases were identified in two other Australian states, genotype D5 in the Northern Territory and genotype D3 in Western Australia. Soon after the g3 outbreak, genotype D7 circulated in Victoria, Queensland, and the Northern Territory.

In recent years, vaccination campaigns have been undertaken in East Timor under the guidance of United Nations Children's Fund (UNICEF) and WHO. In October 1999, UNICEF conducted a major immunization campaign in Dili, East Timor, reaching approximately 4,000 children <5 years of age; in March 2000, >45,000 children were vaccinated (9). Nevertheless, from January 2000 to May 2001, a total of 1,479 cases of suspected measles infection were reported in East Timor.

In an industrialized country like Australia, where no apparent circulation of an indigenous MeV strain occurs, the use of epidemiologic surveillance and molecular characterization is important in tracing the source and transmission pathways of MeV imported from areas where the disease is endemic. Apart from expanding our global knowledge of MeV genotypes, molecular characterization is also useful in clarifying epidemiologic links and distinguishing between vaccine-associated and wild-type infection.

Despite the novelty of the circulating MeV genotypes, our findings highlight the need for continued vigilance if the virus is to be eradicated. The humanitarian movement of refugees from a country where MeV infection is uncontrolled to countries with relatively high MeV herd immunity is now a common occurrence. Thus, the potential for transmission of this highly infectious virus to residual susceptibles in the wider community remains a distinct possibility, as demonstrated by this outbreak. Measures, such as vaccination of military personnel and support staff working directly with displaced persons; use of appropriate infection-control procedures when attending sick refugees; and screening of newly arrived refugees from measles-endemic areas, are likely to decrease transmission of MeV in industrialized countries.
